# Draft genome sequence of *Bacillus safensis* 2T-2, isolated from drinking water

**DOI:** 10.1016/j.dib.2025.111870

**Published:** 2025-07-09

**Authors:** Cornelius C. Bezuidenhout, Lesego G. Molale-Tom, Rinaldo K. Kritzinger, Oluwaseyi Samuel Olanrewaju

**Affiliations:** Unit for Environmental Sciences and Management, North-West University, Potchefstroom Campus, Private Bag X6001, 2520, Potchefstroom, South Africa

**Keywords:** Whole genome sequencing, *Bacillus safensis*, Antibiotic resistant genes, Water treatment

## Abstract

*Bacillus safensis* 2T-2 was isolated from potable water at a municipal water treatment facility in the North West province of South Africa, representing the first report of this species in treated drinking water systems. Whole genome sequencing revealed a 3.78 Mb genome with 41.3 % GC content and 4000 coding sequences distributed across 126 contigs. Genome analysis identified six antibiotic resistance genes, including vancomycin resistance genes (*vanT, vanY*), fosfomycin resistance (*fosBx1*), chloramphenicol resistance (*cat86*), and two disinfectant resistance genes (*qacG, qacJ*). Despite the presence of resistance genes, PathogenFinder analysis confirmed low pathogenic potential (0.168 probability). The strain demonstrated significant biosynthetic capabilities with 12 secondary metabolite gene clusters, including antimicrobial compound production (plantazolicin), biosurfactants (lichenysin), siderophores (bacillibactin, schizokinen), and the lipopeptide fengycin. Five bacteriocin gene clusters were identified, containing three core peptide genes (UviB, plantazolicin, pumilarin) with associated modification and transport genes. Phylogenetic analysis positioned strain 2T-2 closest to *B. safensis* F0–36b, confirming species identification. These findings highlight the dual nature of environmental bacteria in water systems, possessing both concerning antibiotic resistance traits and beneficial biotechnological potential, emphasizing the need for enhanced water treatment strategies while revealing opportunities for bioactive compound discovery.

Specifications Table

**Table:** 

Subject	Biology
Specific subject area	Microbiology, Microbial Genomics, Molecular Biology
Type of data	Raw and analyzed whole genomic sequence data represented by Tables and Figures
Data collection	Bacterial strain was isolated from potable water on nutrient agar at 37 °C. Genomic DNA was extracted using chemagic DNA bacteria kit (PerkinElmer). DNA libraries were prepared using Nextera XT kit and sequenced on Illumina MiSeq 300 platform with paired-end reads (2 × 300 bp). Raw reads were quality-checked with FastQC, trimmed using Trimmomatic, and assembled using SPAdes. Genome annotation was performed using RAST pipeline, followed by identification of antibiotic resistance genes (CARD database), secondary metabolites (antiSMASH), mobile genetic elements (mobileOG-db), and phylogenetic analysis (TYGS).
Data source location	Unit for Environmental Sciences and Management, North-West University, Potchefstroom Campus, Private Bag X6001, 2520, Potchefstroom, South Africa
Data accessibility	This Whole Genome Shotgun project has been deposited at DDBJ/ENA/GenBank under the accession JBNJOE000000000. The version described in this paper is version JBNJOE010000000. Direct URL to data: https://www.ncbi.nlm.nih.gov/nuccore/JBNJOE000000000 Bioproject: PRJNA1247746 Biosample: SAMN47828673

## 1 Value of the Data

• The data is valuable for understanding the safety of potable water by providing insight into the genome of *B. safensis* 2T-2 isolated from the water treatment plant.
• The identification of antibiotic-resistant and virulence genes in *B. safensis* 2T-2 is important in epidemiological surveys and understanding the need for public safety.
• The presence of six antibiotic resistance genes in bacteria surviving current water treatment processes highlights the need for enhanced strategies to prevent resistance gene dissemination through water distribution systems.
• The high‐quality draft genome, supported by deep sequencing coverage (153x), high completeness (99.59 %), and low contamination (1.64 %), provides a robust framework for pan-genome and evolutionary analyses.
• The detection of secondary-metabolite clusters offers a rich source for new bioactive compounds

## 2 Background

*Bacillus safensis* (*B. safensis*) is a Gram-positive, spore-forming bacterium recognized for its versatility and diverse metabolic capabilities [1]. *B. safensis*, originally isolated from spaceship cleanrooms [2], has been detected in diverse environmental niches [3,4]. This study represents the first report of *B. safensis* in treated drinking water. While extensive research has characterized *B. safensis* from spacecraft and terrestrial environments [5,6], its presence and genetic characteristics in water treatment systems remain unexplored. This knowledge gap is critical given the increasing concern over antibiotic resistance proliferation through water distribution networks and the potential biotechnological applications of stress-resistant bacteria in water treatment enhancement. The emergence of antibiotic-resistant bacteria in treated water systems poses significant public health risks, as these environments serve as reservoirs for resistance gene dissemination [7,8]. Genomic characterization of bacteria surviving water treatment processes is essential for understanding treatment efficacy limitations and developing enhanced disinfection strategies. Its resilient physiology enables survival and function in adverse settings, making it a viable choice for environmental and biotechnological applications. Genome mining has identified genes in the production of antimicrobial chemicals, biosurfactants, and enzymes relevant to pollutant breakdown [9,10].

This dataset focuses on a *B. safensis* strain obtained from treated drinking water, providing extensive genomic and functional annotations relevant to water treatment and purification. This includes information on biosurfactant generation, which aids in the remediation of organic contaminants. The data are pertinent for researchers examining biological methods for sustainable water management, as *B. safensis* and similar *Bacillus* species have shown the capacity to enhance the efficacy of biological treatment procedures and support water reuse initiatives [3,11]. This genomic analysis aims to: (i) characterize the antibiotic resistance profile of *B. safensis* in treated water, (ii) identify secondary metabolite production capabilities relevant to water treatment applications, and (iii) provide baseline genomic data for monitoring microbial communities surviving current treatment protocols.

## 3 Data Description

**Fig. 1:**
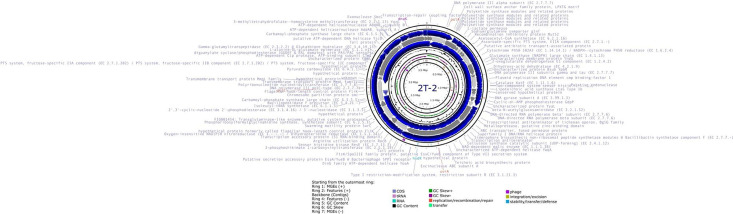
Circular visualization of *B. safensis* 2T-2 genome generated using Proksee. Concentric tracks from outermost to innermost show: (1) mobile genetic elements on forward strand, (2) forward strand coding sequences (blue), (3) contig boundaries, (4) reverse strand coding sequences, (5) GC content variation (black line), (6) GC skew analysis (green/purple), and (7) mobile genetic elements on reverse strand. The 3.78 Mb genome is distributed across 126 contigs with 41.3 % GC content.

We present the whole genome sequence of *B. safensis* 2T-2. We conducted a comprehensive genome mining approach to identify mobile genetic elements, phages, antibiotic-resistant genes, metabolic pathways, and the presence of CRISPR genes. Additionally, we performed phylogenomic analysis to determine evolutionary relationships. The circular representation of the genome with the annotation by RAST is presented in Fig. 1. The assembled genome spans 3.78 Mb, distributed across 126 contigs with a GC content of 41.3 % (Table 1). The genome is 99.59 % complete with low contamination of 1.64 % (Table 1).

**Table 1:** Genome assembly and annotation statistics for *B. safensis* 2T-2 isolated from potable water.

Parameter	Value
Bioproject no.	PRJNA1247746
Biosample no.	SAMN47828673
GenBank accession no.	JBNJOE000000000
SRA accession no.	SRR33016476
Genome size	3785,440 bp
Genome coverage	153 x
Completeness	99.59 %
Contamination	1.64 %
No. of contigs	126
N50	230,017 bp
L50	5
No. of coding sequences	4000
No. of RNAs	80
GC content	41.3 %
No. of subsystems	327

**Fig. 2:**
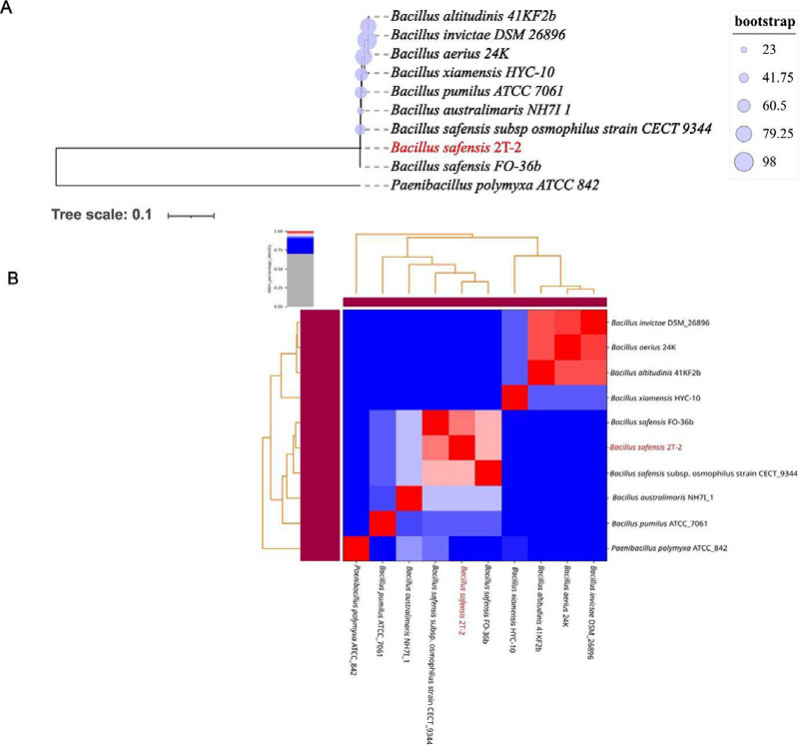
A. Phylogenetic analysis of *B. safensis* 2T-2. The tree shows relationships between 2T-2 and closely related *Bacillus* species, with *B. safensis* F0–36b as the nearest neighbor. Ultrafast bootstrap support values (1000 replicates) are indicated by node circles, with circle size proportional to support value as shown in the legend. The tree was rerooted using *Paenibacillus polymyxa* ATCC 842 as an outgroup. Isolate *2T-2* is highlighted in red. Visualization was performed in iTOL v7 (https://itol.embl.de/). B. Heatmap of Average Nucleotide Identity (ANI) values of representative Bacillus species used in the phylogenetic tree.

Whole-genome taxonomic analysis using GTDB confirmed the bacterium’s identity as *Bacillus safensis*. While *B. safensis* F0–36b was the closest neighbor in a whole genome phylogenetic tree (Fig. 2A) and further confirmed by the average nucleotide analysis (Fig. 2B).

### 3.1 Antibiotic resistance genes prediction

PathogenFinder analysis indicated low pathogenic potential for B. safensis 2T-2 (probability score: 0.168). CARD database analysis revealed six antibiotic resistance genes within the genome. These included vancomycin resistance genes (*vanT* and *vanY*) from the vanG cluster, fosfomycin resistance gene (*fosBx1*), and two disinfectant resistance genes (*qacG* and *qacJ*) (Table 2).

**Table 2:** Antibiotic resistance genes identified in the *B. safensis* 2T-2 genome using the Comprehensive Antibiotic Resistance Database (CARD) with the RGI tool. Genes are categorized by resistance mechanism, target antibiotic class, and gene family according to CARD classification criteria.

RGI Criteria	ARO Term	AMR Gene Family	Drug	Resistance Mechanism
Strict	*vanT* gene in vanG cluster	glycopeptide resistance gene cluster, vanT	glycopeptide antibiotic	antibiotic target alteration
Strict	*Bacillus pumilus* cat86	chloramphenicol acetyltransferase (CAT)	phenicol antibiotic	antibiotic inactivation
Strict	*qacJ*	small multidrug resistance (SMR) antibiotic efflux pump	disinfecting agents and antiseptics	antibiotic efflux
Strict	*qacG*	small multidrug resistance (SMR) antibiotic efflux pump	disinfecting agents and antiseptics	antibiotic efflux
Strict	*FosBx1*	fosfomycin thiol transferase	phosphonic acid antibiotic	antibiotic inactivation
Strict	*vanY* gene in vanG cluster	*vanY*, glycopeptide resistance gene cluster	glycopeptide antibiotic	antibiotic target alteration

### 3.2 Secondary metabolites and biosynthetic gene cluster identification

**Fig. 3:**
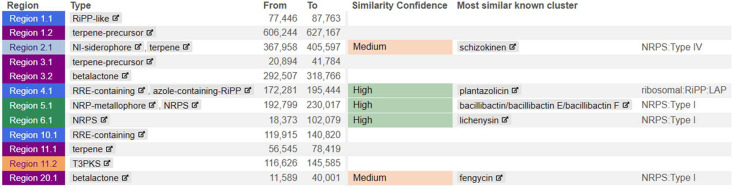
Secondary metabolite biosynthetic gene clusters identified in *B. safensis* 2T-2 using antiSMASH analysis. The genome contains 12 clusters, including NRPS genes for siderophore production (bacillibactin), biosurfactant synthesis (lichenysin), lipopeptide production (fengycin), RiPP genes for antibiotic synthesis (plantazolicin), and siderophore schizokinen production. Each colored region represents a different biosynthetic gene cluster, with the predicted secondary metabolite type indicated.

**Fig. 4:**
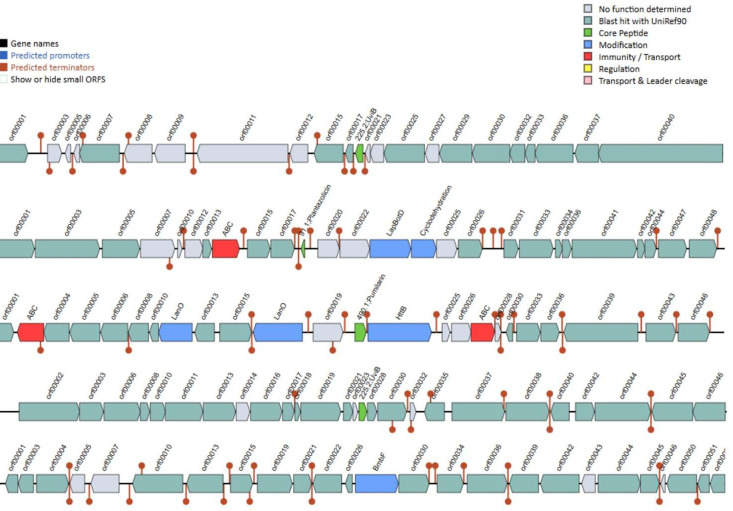
Bacteriocin gene clusters identified in *B. safensis* 2T-2 using BAGEL4 analysis. Five gene clusters were detected, containing 3 core peptide genes (UviB, plantazolicin, and pumilarin), 5 modification genes involved in post-translational processing, and 1 immunity and transport gene for self-protection and secretion. Gene arrangements and functional annotations are shown with directional arrows indicating transcription orientation.

*B. safensis* 2T-2 harbored 12 biosynthetic gene clusters, including genes encoding siderophore schizokinen synthesis, a RiPP gene cluster for the antibiotic plantazolicin, and three NRPS gene clusters responsible for producing the siderophore bacillibactin, biosurfactant lichenysin, and lipopeptide fengycin (Fig. 3). BAGEL4 identified five gene clusters of interest related to the synthesis of secondary metabolites, including 3 core peptide genes (UviB, plantazolicin, and pumilarin), 5 modification genes, and 1 immunity and transport gene (Fig. 4).

## 4 Experimental Design, Materials, and Methods

The *B. safensis* 2T-2 was isolated from potable water at a municipal water treatment facility in the North West Province of South Africa. Strain was isolated on nutrient agar at 37 °C for 24 h. Pure isolate was obtained after further subculturing on nutrient agar at 37 °C for 24 h. DNA from pure culture was extracted using the chemagic DNA bacteria kit (PerkinElmer, Germany), following the manufacturer’s protocol. The DNA quantity and quality were assessed using a NanoDrop 2000 UV–Visible spectrophotometer (Thermo Fisher Scientific). DNA libraries were prepared using the Nextera XT kit (Illumina Inc., San Diego, CA) according to the instructions provided by the manufacturer and sequenced using the Illumina MiSeq 300 platform with paired-end reads at the North-West University sequencing facility. The generated raw paired-end fastq reads (2  ×  300 bp) were quality checked using FastQC v.0.11.7 [12]; low-quality bases were trimmed using Trimmomatic v.0.39 [13], and the data quality was rechecked using FastQC v.0.11.7 [12]. The cleaned reads were assembled using SPAdes v.3.15.5 [14]. QUAST v.5.0.2 [15] was used to evaluate the quality of the genome assembly. The completeness and contamination were assessed using CheckM v.1.1.6 [16]. The assembled draft genome was annotated using the Rapid Annotations using Subsystems Technology (RAST) pipeline [17]. The annotated genomes were assessed against the Genome Taxonomy Database (GTDB) [18] using GTDB-Tk software v.1.7.0 [19]. The circular plot of the genome was created using proksee [20] using the RAST annotated file as input. For phylogenetic analysis, the draft genome of isolate 2T-2, along with reference Bacillus genomes and Paenibacillus polymyxa (used as the outgroup), was analyzed using GTDB-Tk v2.3.2 [19] to generate a concatenated alignment of 120 bacterial single-copy marker genes. The alignment was subsequently used to infer a maximum-likelihood tree in IQ-TREE v2.4.0 [21] with ModelFinder Plus (-m MFP) and 1000 ultrafast bootstrap replicates (-B 1000). The resulting consensus tree was rerooted on P. polymyxa using Gotree v0.4.2 [22]. Antibiotic resistance genes were identified using the RGI tool on the CARD database [23], phages were detected with the phastest tool [11], CRISPR-Cas systems were detected using the CRISPRFinder tool [24], secondary metabolites were detected using the online platform Antibiotics and Secondary Metabolites Analysis Shell (antiSMASH) v.8.0 [25], bacteriocins were detected using the web-based version of Bagel4 [26], mobile genetic elements were identified using the mobileOG-db tool [27], and pathogenicity of the genome was identified using PathogenFinder v.1.1 [28] with the parameters “firmicutes and Assembled Genome/Contig”. Default parameters were used for all software and tools except where otherwise noted.

## Limitations

Not applicable

## Ethics Statement

The authors have read and followed the ethical requirements for publication in Data in Brief and confirm that the current work does not involve human subjects, animal experiments, or any data collected from social media platforms.

## CRediT Author Statement

**Oluwaseyi Samuel Olanrewaju:** Conceptualization, Data curation, Formal analysis, Methodology, Software, Visualization, Writing – original draft, Writing – review & editing. **Lesego G. Molale-Tom:** Conceptualization, Investigation, Project administration, Supervision, Writing – review & editing. **Rinaldo K. Kritzinger:** Conceptualization, Investigation, Methodology. **Cornelius C. Bezuidenhout:** Conceptualization, Funding acquisition, Resources, Supervision, Writing – review & editing.

## Data Availability

NCBI GenbankBacillus safensis strain 2T-2, whole genome shotgun sequencing project (Original data).
